# The Burden of ABO-Incompatible Kidney Transplantation: Readmission Rates and Complications, a Twenty-Year Analysis

**DOI:** 10.3390/jcm13237477

**Published:** 2024-12-09

**Authors:** Caroline Berchtold, Kerstin Huebel, Fabian Roessler, Nicole Graf, Philipp Dutkowski, Kuno Lehmann, Thomas Mueller, Olivier de Rougemont

**Affiliations:** 1 Department of Visceral Surgery and Transplantation, University Hospital Zurich, 8091 Zurich, Switzerland; kerstin.huebel@usz.ch (K.H.); fabian.roessler@usz.ch (F.R.); philipp.dutkowski@usb.ch (P.D.); kuno.lehmann@spital.so.ch (K.L.); tfmueller1492@hotmail.com (T.M.); olivier.derougemont@hin.ch (O.d.R.); 2 Independent Researcher, 8403 Winterthur, Switzerland; graf@biostatistics.ch

**Keywords:** ABO incompatible, kidney, transplantation, burden, hospitalization, paired donation

## Abstract

**Background/Objectives**: ABO-incompatible live-donor kidney transplantation (ABOi-LDKT) has become an established treatment for end-stage renal disease. Non-inferiority in the long-term graft function compared to ABO-compatible live-donor kidney transplantations (ABOc-LDKTs) has been shown. However, the assumed burden due to complications owing to increased immunosuppression inherent to ABOi-LDKTs has not yet been quantified. The aim of this study was to determine if ABOi-LDKT recipients suffer from additional morbidity and whether the resulting burden is justified. **Methods**: We retrospectively analyzed 45 matched pairs of ABOi-LDKTs and ABOc-LDKTs transplanted over a twenty-year period from January 2000 to March 2020. The number and duration of postoperative readmissions, surgical complication rates according to Clavien–Dindo and its comprehensive complication index (CCI), kidney function, occurrence of new-onset diabetes, and infections as well as tumor incidence were analyzed. **Results**: Patient and graft survival, as well as graft function, were comparable between the two groups. There were no significant differences in terms of complications, readmission rates, and length of readmission, as well as infection and rejection rates. The median CCIs for ABOi-LDKTs and ABOc-LDKTs at primary discharge and 3, 6, 12, and >12 months were 20.9 vs. 20.9 (*p* = 0.363), 31.4 vs. 33.7 (*p* = 0.438), 33.7 vs. 33.7 (*p* = 0.875), 20.9 vs. 33.1 (*p* = 0.25), and 27.1 vs. 31.9 (*p* = 0.163), respectively. **Conclusions**: ABOi-LDKT seems safe, with comparable outcome, complication, and readmission rates to ABOc-LDKT. In recipients with ABOi living donors, transplantation should not be delayed solely due to concerns over increased perioperative risks.

## 1. Introduction

Kidney transplantation (KT) is the treatment of choice for patients with end-stage renal disease (ESRD) from a clinical and economic standpoint [1]. Living donor kidney transplantation (LDKT) has better outcomes in terms of patient and graft survival compared to deceased donor kidney transplantation (DDKT) [2,3].

However, when an ABO-compatible (ABOc) living donor may not be available, ABO-incompatible kidney transplantation (ABOi-LDKT) represents a good chance to increase the living donor pool. With the evolution of desensitization protocols and improved immunosuppression regimens in the past decades, favorable graft and patient survival rates and non-inferiority to blood group-compatible KT have been reported [4]. ABOi-LDKT currently makes a substantial and growing contribution to LDKT. However, one drawback of ABOi-LDKTs is that more potent immunosuppression, including desensitization, is required, bearing the risk of increased complication rates, namely infections and hemorrhage [4,5,6]. An increased risk for malignancy is pathophysiologically expected; however, this has not yet been shown [7]. This may result in additional patient burden, in terms of prolonged hospital stay or emergency readmission. Up to one-third of patients after KT are readmitted on an emergency basis [8] and such readmissions are associated with adverse outcomes, including graft loss and increased mortality [8,9]. Moreover, hospitalizations have a socioeconomic and psychological impact on society and the patient. However, whether concerns on higher readmission rates and extended hospitalization following ABOi-LDKT are justified and to what extent has not yet been well quantified [10].

An alternative to ABOi-LDKT for recipients and their ABOi donating partner is to sign up for a kidney paired donation (KPD) program and thereby become an ABOc-LDKT recipient. Such programs have been successfully implemented in many countries and can help optimize immunological matching. This, however, often results in longer waiting times, thereby risking inferior outcomes after transplantation [11].

The aim of the study was to determine if ABO-incompatible recipients have additional morbidity and whether this burden, defined as readmissions and complications, is justified or whether kidney paired donation programs should be advocated for.

## 2. Materials and Methods

### 2.1. Study Population

We retrospectively analyzed data from all patients who underwent a KT between January 2000 and March 2020. A total of 1191 KTs were performed at our center during this period, where 540 were LDKTs. In total, 46 patients underwent ABOi-LDKTs.

The primary outcome was to determine whether ABOi-LDKTrs experience an additional burden of hospital readmissions.

Recipients of an ABOi-LDKT were hospitalized approximately one week prior to surgery for immunoadsorption. Recipients of an ABOc-LDKT were hospitalized two days prior to surgery for a final check-up.

Out of 46 ABOi and 482 ABOc recipients, a total of 45 ABOi recipients could be matched with 45 ABOc recipients. Propensity scores were estimated via logistic regression. The following covariates were included: sex, inherent pathophysiological probability for disease recurrence, duration of dialysis, age at transplantation, and year of transplantation. All covariates are known to influence outcomes after transplantation. The covariate ‘year of transplantation’ was included to compare outcomes of patients subjected to the most similar protocols and medical standards as well as to minimize the impact of skill variability in surgeons. A 1:1 match was performed without replacement by using a combined exact (sex, inherent pathophysiological probability for disease recurrence, and duration of dialysis) and nearest neighbor matching algorithm with calipers on age at transplantation and year of transplantation. The balance was inspected both numerically and visually. Standardized differences between both groups of no more than 10% were accepted. Data were extracted out of the tertiary care ‘in-house’ encrypted digital patient database from routine follow-up documentation or documentation of readmission after transplantation. Admissions to other hospitals were included if data were present in the patient file. Given the size and geographical and infrastructural characteristics of Switzerland, graft recipients are, with very few exceptions, treated at their transplantation center. Therefore, no geographic variable was included as a covariate.

### 2.2. Institutional Review Board Approval

This study was approved by the local ethics committee (project number 2020-01-499).

#### 2.2.1. Protocol for ABO-Incompatible Kidney Transplantation

The desensitization starts one month preoperatively with a single dose of intravenous Rituximab. There is no titer threshold for ABOi transplantation. Immunoadsorption is performed if blood group antibody titers are ≥1:8. Transplantation is performed as soon as titers are <1:8. Induction therapy with Basiliximab is administered on days 0 and 4 after transplantation. Basic immunosuppression consists of Tacrolimus, Mycophenolate Mofetil, and steroids. Tacrolimus and Mycophenolate Mofetil start one week prior to transplantation with dose adjustments according to blood level measurements. In the case of adsorption treatment, oral steroids are initiated simultaneously. Alternatively, high-dose steroids are started on day 0 through day 6, followed by tapering every 14 days until a maintenance dose of 5 to 7.5 mg is achieved. Protocol biopsies are performed on day zero, three months, and one year after transplantation. According to the Swiss ABOi protocol, blood group antibody titers are measured in all patients daily for 2 weeks, weekly until day 31, and 3, 6, and 12 months thereafter.

#### 2.2.2. Readmission Rate

Readmissions were defined as unplanned hospital stays after transplantation. Given the different time spans for each patient (date of transplantation—March 2020) the number of all readmissions of ABOi-LDKTrs was divided by the cumulative number of years analyzed in ABOi-LDKTr. Similar calculations were performed for ABOc-LDKTr. The time spans analyzed and compared were defined from primary discharge to 90 days postoperatively, from 90 days to 6 months postoperatively, from 6 to 12 months postoperatively, and from 1 year postoperatively to March 2020.

#### 2.2.3. Complications

We assessed complications at primary discharge and during the time spans of primary discharge to 3 months, 3–6 months after transplantation, 6–12 months after transplantation, and hospitalizations during the time >12 months (CCIs were calculated separately for each hospitalization) until March 2020 according to the validated Clavien–Dindo classification [12,13]. To assess cumulative postoperative morbidity, we used the comprehensive complication index (CCI), a metric of morbidity developed to integrate and numerically represent all postoperative complications graded according to the Clavien–Dindo classification. The CCI ranges from 0 (no complication) to 100 (death) [14,15,16].

#### 2.2.4. Donor-Specific Antibodies

The presence of DSA was defined at MFI > 1000.

#### 2.2.5. Graft Function

Kidney transplant function was assessed according to the Chronic Kidney Disease Epidemiology Collaboration (CKD-EPI) equation with creatinine values used to calculate the glomerular filtration rate (GFR) [17,18].

#### 2.2.6. Rejection

Only biopsy-proven rejection episodes were rated.

#### 2.2.7. Data Collection

After completing the matching process, the following parameters were extracted from patient files of all matched pairs: rate of readmissions relative to patient-years analyzed; surgical complication rate according to Clavien–Dindo classification [12,13]; comprehensive complication index (CCI) [14,15,16] during primary and subsequent hospitalizations during the time spans of primary discharge to 3 months, 3–6 months after transplantation, 6–12 months after transplantation, and hospitalizations during the time >12 months (CCIs were calculated separately for each hospitalization) until March 2020; delayed graft function (DGF, defined as the necessity for dialysis within the first week after transplantation); length of hospital stay including preoperative hospitalization duration; kidney function; and graft and patient survival at 1, 5, and 10 years after transplantation as well as the rate of rejection. There were no protocol readmissions.

#### 2.2.8. Statistical Analysis

The goal was to test a non-inferiority hypothesis for the primary endpoint of the number of readmissions per year. The non-inferiority margin was defined as an increase in the hospitalization rate of ≤20%. Despite the study not being powered to test this hypothesis given the limited number of ABOi-LDKTs, we assessed the rate ratio with corresponding 95% confidence intervals. The primary endpoint was thus compared between the matched pairs with a Poisson regression model. A generalized estimating equation (GEE) approach with an independent correlation structure was used to account for the matched pairs. For binary outcomes such as graft rejection, DGF, hospitalization due to infection, tumor incidence, and NODAT, the matched pairs were compared with a McNemar test with continuity correction. For numerical outcomes such as length of hospital stay, surgical complication rate according to Clavien–Dindo, or renal function, a Wilcoxon signed-rank test with continuity correction was applied (median [IQR]). The survival functions (patient and graft survival) were compared with the Akritas test (Akritas, 2009). The Akritas test assigns a score to each member of a pair based on rank-transformed survivor functions and conducts a paired *t*-test for these scores. All analyses were performed in the R programming language (version 4.1.0) (R Core Team, 2021).

## 3. Results

### 3.1. Study Population

Patient characteristics are shown in Table 1. Standardized differences between both groups of no more than 10% were accepted which led to the exclusion of one case and 45 matched pairs. All 45 matched transplantations were first kidney transplantations. The median follow-up time was 100 months (range 2–175 months). No patient was excluded from the final analysis. The average donor age was 51.8 and 52 years for ABOc and ABOi patients, respectively.

### 3.2. Readmissions

The overall readmission rate was 0.599 per year for ABOi-LDKT and 0.419 per year for ABOc-LDKT. ABOc recipients were readmitted 156 times in 372.7 cumulative person-years (date of transplantation—March 2020 for each patient). ABOi-LDKTrs were readmitted 213 times in 355.7 cumulative person-years. From discharge until 3 months after transplantation, ABOi-LDKTrs were hospitalized 18 times in total, and ABOc-LDKTrs were hospitalized 30 times. During the time span of 3 to 6 months after transplantation, both ABOi-LDKT and ABOc recipients were readmitted 13 times in total. During the period of 6 to 12 months post-transplantation, both ABOi-LDKT and ABOc-LDKTrs were hospitalized 22 times in total. For the time span from 1 year after transplantation until March 2020, ABOi-LDKTrs were readmitted 160 times in 313.7 patient-years corresponding to a rate of 0.510 per year, whereas ABOc-LDKTrs were readmitted 91 times in 328.7 patient-years corresponding to a rate of 0.277 per year. The overall difference in readmissions is not statistically significant.

The median length of readmission was similar for ABOi-LDKT compared to ABOc-LDKT ((13.5 [4.8–39.0]) versus (10.0 [3.0–33.0]), (*p* = 1)). Infection was the most common single cause of readmission during 3, 6, 12, and >12 months post-transplantation. Among these, urinary tract infections with *Escherichia coli* (*E. coli*) were the most common.

### 3.3. Perioperative Hospitalization Duration

Postoperative hospitalization duration differed; however, it did not differ significantly (*p* = 0.519), with 9.5 [8.0, 12.5] days for ABOi-LDKT and 9.0 [7.0, 11.0] days for ABOc-LDKTr.

Prior to transplantation, ABOi-LDKT patients were hospitalized for 10.0 [8.0, 13.0] days, compared to just 2.0 [2.0, 2.0] days for ABOc-LDKTrs (*p* < 0.001).

### 3.4. Kidney Function

Kidney function was comparable for ABOc-LDKT and ABOi-LDKT grafts. Solely one year after transplantation, the median eGFR was significantly higher for ABOi-LDKT (*p* = 0.004) (Table 2). In both groups, there was one case of DGF.

### 3.5. Patient and Graft Survival

**Figure 1:**
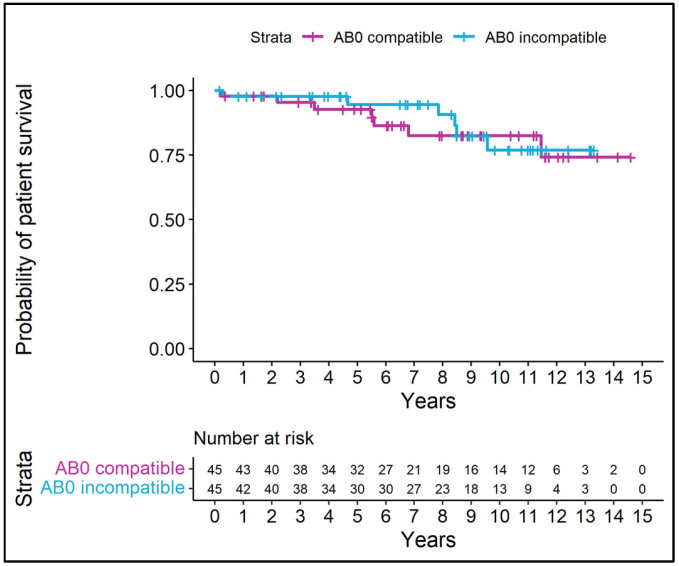
Probability of patient survival: Kaplan–Meier curves for time to death. Vertical dashes indicate censored data.

**Figure 2:**
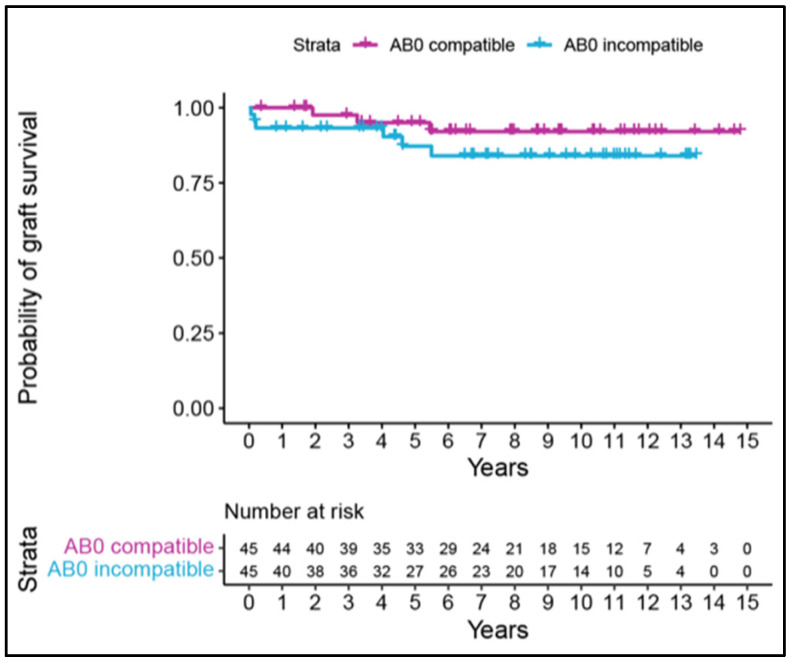
Probability of graft survival: Kaplan–Meier curves for time to graft loss. For each patient not known to have died, graft survival is censored at the time of the last date known to be alive.

Patient survival for ABOc-LDKTrs and ABOi-LDKTrs was comparable (*p* = 0.683). Within the observation period, seven ABOc-LDKTrs and six ABOi-LDKTrs died (*p* = 0.683), (Figure 1). The causes of mortality were septic shock (in one ABOc-LDKTr and four ABOi-LDKTrs), subarachnoid bleeding and spinocellular carcinoma (one each in one ABOc-LDKTr), gastrointestinal bleeding (in one ABOi-LDKTr). In four ABOc-LDKTrs and one ABOi-LDKTr, the cause of death remained unknown. Death-censored graft survival for ABOc-LDKT and ABOi-LDKT was comparable (*p* = 0.221), (Figure 2). Four of six graft losses in ABOi-LDKTs were associated with infections, whereas infection was never causative for or preceded graft loss in ABOc-LDKT, corresponding to 66.67% in ABOi vs. 0% in ABOc. In our cohort, out of six graft losses in ABOi-LDKTrs, only one presented preformed class I and II donor-specific antibodies (DSAs), and none of the three graft losses in ABOc-LDKTrs presented class I and II DSAs. Preformed class I or II DSAs occurred in a total of nine ABOi-LDKTrs and eight ABOc-LDKTrs. The highest blood group antibody titers in patients before starting immunoadsorption were 1:1024 IgG and 1:256 IgM. Median HLA mismatches were found in four ABOc-LDKTrs and five ABOi-LDKTrs.

### 3.6. Postoperative Complications

#### 3.6.1. CCI

The median CCI to discharge after transplantation was 20.9 [0.0–33.5] and 20.9 [0.0–30.8] for ABOi-LDKTrs and ABOc-LDKTrs, respectively (*p* = 0.363).

CCIs for readmissions were assessed separately (non-cumulative) for each readmission and can be found in Table 3.

#### 3.6.2. Major Complications (Clavien–Dindo Classification ≥ 3a)

The highest graded complication for each rehospitalization during the entire time analyzed (ABOi-LDKT 355.7 patient-years; ABOc-LDKT 372.7 patient-years) was identified. Complications graded 3a (requiring surgical, endoscopic, or radiologic intervention, not under general anesthesia) were found 43 times in ABOc-LDKTrs and 50 times in ABOi-LDKTrs; these complications occurred in a total of 22 ABOc-LDKTrs and 15 ABOi-LDKTrs, respectively.

Complications graded 3b (requiring surgical, endoscopic, or radiologic intervention, under general anesthesia) were identified 44 times in ABOc-LDKTs and 61 times in ABOi-LDKTrs. These complications occurred in 23 ABOc-LDKTrs and 23 ABOi-LDKTrs, respectively. Complications graded 4a (life-threatening complications requiring ICU management with single-organ dysfunction) were found 8 times in a total of seven ABOc-LDKTrs and 11 times in five ABOi-LDKTrs. Complications graded 4b (life-threatening complications requiring ICU management with multi-organ dysfunction) were identified three times in three ABOc-LDKTrs and twice in a single ABOi-LDKTr.

### 3.7. Infection

From discharge to 90 days postoperative, three (6.7%) ABOc-LDKTrs and four (9.1%) ABOi-LDKTrs were hospitalized due to infection (*p* = 1).

From 3 to 6 months postoperative, one (2.3%) ABOc-LDKTrs and five (11.6%) ABOi-LDKTrs were hospitalized due to infection (*p* = 0.221). From 6 to 12 months postoperative, 11 (25%) ABOc-LDKTrs and 10 (23.8%) ABOi-LDKTrs were hospitalized due to infection (*p* = 1).

After the first year of transplantation up to March 2020, 22 (50%) ABOc-LDKTrs and 23 (53.3%) ABOi-LDKTrs were hospitalized due to infection (*p* = 1).

For every period analyzed, *E. coli* was the most reported germ identified as causative for rehospitalization. Herpes simplex virus, Cytomegalovirus, Candida, and Aspergillus infections were further infectious agents with no statistically significant predominance in either ABOi-LDKTrs or ABOc-LDKTrs.

### 3.8. Rejection

In total, 11 ABOi-LDKTrs (25.6%) and 17 ABOc-LDKTrs (37.8%) had an acute rejection. For the McNemar test, only the discordant pairs (matched patients in which either the ABOi-LDKTr or the ABOc-LDKTr suffered from a rejection) were considered, i.e., 5 (ABOi-LDKTr) versus 11 (ABOc-LDKTr) (*p* = 0.211). In 21 pairs, neither the ABOi-LDKTr nor the ABOc-LDKTr suffered from rejection, and in 6 matched recipients, both the ABOi-LDKTrs and the ABOc-LDKTrs suffered from rejection. In total, six rejections were antibody-mediated, with five in ABOc-LDKTr- and one in an ABOi-LDKTr. A total of 17 rejections were T-cell mediated with 9 in ABOc-LDKTrs and 8 in ABOi-LDKTrs. Three rejections were both antibody- and T-cell-mediated with two occurring in ABOc-LDKTrs and one occurring in an ABOi-LDKTr. One ABOi and one ABOc transplant showed borderline changes.

Graft rejection within the first 90 days was the reason for hospitalization in two (4.4%) ABOc-LDKTrs and one (2.3%) ABOi-LDKTr (*p* = 1). From 3 to 6 months after transplantation, one (2.3%) ABOc-LDKTr and two (4.7%) ABOi-LDKTrs were admitted for treatment of graft rejection (*p* = 1). From 6 months to 1 year after transplantation, two (4.7%) ABOc-LDKTrs and one (2.4%) ABOi-LDKTr were admitted for treatment of graft rejection and from 1 year post-transplantation to March 2020; four (9.1%) ABOc-LDKTrs and two (4.8%) ABOi-LDKTrs were admitted for treatment of graft rejection (*p* = 1).

### 3.9. Tumor Incidence

A total of seven ABOi-LDKTrs were diagnosed with 11 cancer types in 355.7 cumulative person-years (date of transplantation—03/2020). A total of 12 ABOc-LDKTrs were diagnosed with 15 cancer types in 372.7 cumulative person-years. The most common diagnosis was spinocellular carcinoma. No difference in tumor incidence for ABOi-LDKTrs and ABOc-LDKTrs was detected.

### 3.10. NODAT

Within the first three months after transplantation, the prevalence of NODAT was significantly higher in ABOi-LDKTrs (14; 31.8%) vs. ABOc-LDKTrs (3; 6.7%) (*p* = 0.003); however, the difference stalled and became insignificant from six months after transplantation onwards.

### 3.11. Cardiovascular Events

Cardiovascular events occurred in isolated cases only presenting with ACS, which was diagnosed in one ABOc-LDKTr and one ABOi-LDKTr at <3 months after transplantation and in three ABOc-LDKTrs and two ABOi-LDKTrs > 1 year after transplantation.

## 4. Discussion

This is, to our knowledge, the first study analyzing the ‘burden’ of hospital readmission, complication rate, and severity in ABOi-LDKT compared to ABOc-LDKT [19]. In our comparative analysis of two highly matched cohorts, we did not find a significant negative impact of ABOi-LDKT on readmission rates, postoperative complications, or length of hospital stay. Overall, our results suggest no significant negative impact of ABO incompatibility on postoperative outcomes, and hence no additional ‘burden’ of ABOi-LDKT was detectable in our cohort.

Data on postoperative complications after ABOi-LDKT are conflicting [20,21]. Interestingly, it seems that the intensified immunosuppressive regimen does not automatically increase the rate of complications. On the other hand, data on readmission rates after ABOi-LDKT are lacking.

In our cohort, the length of hospitalization was longer for recipients of an ABOi-LDKT; however, it was not statistically significant. This was expected due to measurements of antibody titers following ABOi-LDKT on a regular basis. The same is true for preoperative hospitalization, due to the extended immunosuppression and immunoadsorption prior to transplantation.

CCI was not significantly higher in ABOi-LDKTr. Notably the number of times a cer- 309 tain grade of complication occurred was with one exception (grade 4a), persistently higher 310 in ABOi-LDKTr. However, the number of ABOi-LDKTrs concerned compared to ABOc-LDKTrs was consistently lower. This suggests that compared to ABOc-LDKTrs, overall, ABOi-LDKTrs suffered from more complications; however, this result was found in a lower number of recipients.

Graft survival in our cohort was excellent. Kidney function in ABOi-LDKT was consistently higher at any point in time after transplantation compared to ABOc-LDKT; however, this was only statistically significant after one year. This could be due to the intensified immunosuppressive regimen, reflected by a lower rate of graft rejections in ABOi-LDKT. However, the difference in rejections was not statistically significant, corresponding to previous analyses [22,23,24]. Rejection may have been found in protocol biopsies independently of symptoms or graft function. Overall, rejection led to very few hospitalizations (52% of all ABOc-LDKTrs and 54% of all ABOi-LDKTrs diagnosed with rejection), and it did not have an impact on graft survival. Therefore, the clinical relevance of the increased number of rejections in ABOc-LDKT is questionable.

Moreover, in our cohort, ABOi-LDKTrs did not have a higher risk for graft loss compared to ABOc-LDKTrs. This is in line with Park W.Y. et al. [22] (graft survival 100% for ABOi-LDKT and ABOc-LDKT), Thukral S. et al. [23] (graft survival 96.67% for ABOi-LDKT and ABOc-LDKT during one-year follow-up), and Opelz G. et al. [24] (graft survival 89.9% for ABOi-LDKT and 90.1% for ABOc-LDKT during three-year follow-up).

However, our results contrast with Massie et al., stating higher rates of graft loss in ABOi-LDKT [25], and Ko et al. [26], with infectious complications being the main cause of early graft loss. Massie et al. conclude that once the first 180 days after ABOi-LDKT have been overcome, long-term patient and graft survival are superior to ABOc-LDKT and patients remaining on the waiting list [25]. The differing outcomes for ABOi-LDKTrs may be due to blood group distribution in recipients, as suggested by de Weerd et al. [27]. Reporting of recipient’s blood groups in ABO-LDKT is not yet consistently achieved; however, its importance has been shown and emphasized as a prerequisite for studies on ABOi-LDKT [27,28]. In our cohort, more than two-thirds (67%) of ABOi-LDKTrs had blood group O, similar to the study by de Weerd et al. [27]; however, we did not confirm inferior outcomes in ABOi-LDKT.

Montgomery et al. [29] detected a statistically increased risk for ABOi-LDKT, but only within the first 14 days after transplantation. Similarly, in our study, three out of six graft losses in ABOi-LDKT occurred within the first three months after transplantation and all were caused by infections. In contrast, in ABOc-LDKT, no graft loss occurred earlier than 23 months after transplantation, and none was due to infections. At our center, for ABOi-LDKT, parallel to apheresis, Tacrolimus and steroids are administered starting a week prior to transplantation. In contrast, immunosuppression in ABOc-LDKT is only started on the day of transplantation. Moreover, in ABOi-LDKT, steroids are applied at a higher dose and tapering occurs slower compared to ABOc-LDKT. In conclusion, two-thirds of graft losses in ABOi are preceded by infection, and the different and intensified immunosuppressive treatment in ABOi-LDKT might be associated with graft loss, even though differences in infection rates were not statistically significant.

Patient survival was unaffected by ABO compatibility and, importantly, no mortality was observed due to surgical complications. This is again in contrast to Ko et al. [26], where patients with both ABO and HLA incompatibilities showed inferior rates of overall patient and graft survival due to infectious complications. Overall, seven ABOc-LDKTrs and six ABOi-LDKTrs died within the study period, with septic shock representing the main cause of death for ABOi-LDKTrs. In our cohort, three out of four deaths due to sepsis in ABOi-LDKTrs occurred 5 to 8 years after transplantation. Similarly, Thukral et al. [23] stated septic shock as the main cause of death in their cohort. Montgomery et al. [29] showed comparable patient survival over an average follow-up of 5 years. In contrast, a European collaborative study found that survival was significantly lower in ABOi-LDKT due to infections in the first year after transplantation [24]. However, this difference stalled over time. Importantly, overall follow-up times vary greatly in these studies. Yet again, infection seems to be a predominant and recurring factor. ABOi-LDKTrs should therefore be evaluated with great vigilance if presenting with signs of infection during the first year after transplantation.

Regarding the incidence of malignant tumors related to immunosuppression, we did not detect any differences over a 20-year period. This is in accordance with results from Opelz et al. [24]. In our cohort, squamous cell carcinomas followed by basal cell carcinomas were the most common tumors, as skin cancer is known to be the most frequently diagnosed malignancy in kidney transplant recipients [30,31].

Before the introduction of the kidney paired donation program, it was common practice at our institution to offer ABOi TPL with all incompatible living donors fulfilling our donation criteria. To overcome incompatibility, kidney paired donation programs have been introduced in many countries. However, in countries with small populations and accordingly small donor pools, this may result in long waiting times and consecutive inferior outcomes after transplantation [11] despite KPD recipients showing comparable outcomes to ABOc-LDKTrs [32]. Although no critical number of pairs has been defined, some suggest that around 100 pairs are a desirable number to start a KPD program [33]. Not only is there excellent national cooperation between different transplant centers, but even international cooperation is currently emerging to increase donor pools. Efforts have been made to build international kidney exchange programs, and global projects are in the early stages [34,35]. Nevertheless, at present, not having found a significant ‘burden’ issuing from ABOi-LDKT, with the donation remaining directed and currently implemented exchange programs being in early stages, we recommend not delaying transplantation due to incompatibility.

This study has some limitations. The comprehensive complication index (CCI) was designed to measure postoperative complications in a reproducible way [16]. In our study, we applied the CCI over the entire time analyzed to generate comparable parameters. However, complications occurring several years after surgery cannot be considered postoperative or directly linked to surgery. Furthermore, the approach of a quantitative analysis of the cumulative post-transplant ‘burden’ for the patients facilitates the comparison between the two treatment strategies. No ethnic or geographical covariate was included for propensity score matching. Graft recipients are instructed to search for treatment at their transplantation center if necessary. Recipients most often adhere to this recommendation due to the small size of Switzerland and its geographical and infrastructural characteristics. Even if patients were hospitalized outside the transplantation center, excellent cooperation between peripheral hospitals and transplantation centers leads to treatment often being guided by the transplantation center, and the respective information is available in in-patient files. Therefore, it is highly unlikely that admissions were missed.

Despite including all patients transplanted at our center from 2000 to 2020, the overall number of matched pairs is rather low in this single-center observational study. Hence, the study is not powered to test the hypothesis of non-inferiority of ABOi-LDKT regarding the number of rehospitalizations or overall burden. Nevertheless, there was a trend towards ABOi-LDKTrs having higher readmission rates. We think the granular matching of the individual pairs as well as standardized comparable protocols for both groups and the monitoring at a singular center provides additional strength to this comparative analysis.

## 5. Conclusions

In conclusion, ABOi-LDKT seems to be a safe option and offers comparable outcomes and readmission rates to ABOc-LDKT. In our analysis, we did not detect any major, additional burden for patients undergoing ABOi-LDKT. ABOi pairs should be informed about ABOi transplantation as soon as the incompatibility is discovered. The option of ABOi-LDKT should not be discarded solely due to concerns about increased complication or readmission rates. Transplantation, whether compatible or incompatible, should not be delayed so as not to worsen the recipient’s health by prolonging dialysis time.

## Figures and Tables

**Table 1:** Propensity-matched baseline covariates and population characteristics.

Matching Variables	ABO Compatible	ABO Incompatible
n	45	45
Male n (%)	29 (64.4)	29 (64.4)
Female n (%)	16 (35.6)	16 (35.6)
Age at KT (mean (SD))	49.5 (10.8)	50.1 (11.3)
Recurrent disease n (%)	12 (26.7)	12 (26.7)
Year of KT (mean (SD))	2011.4 (3.9)	2011.8 (3.9)
0 to <7 months dialysis n (%)	17 (37.8)	17 (37.8)
7 to 24 months dialysis n (%)	15 (33.3)	15 (33.3)
>24 months dialysis n (%)	13 (28.9)	13 (28.9)

**Table 2:** Kidney function eGFR (mL/min per 1.73 m^2^) at 1, 5, and 10 years after transplantation using CKD-epi (median [IQR]).

Variable	ABO Compatible	ABO Incompatible	*p*-Value
eGFR at 1 year	47.5 [41.2, 55.0]	59.0 [47.0, 68.5]	0.004
eGFR at 5 years	50.0 [39.0, 63.0]	60.0 [46.2, 70.0]	0.136
eGFR at 10 years	44.0 [32.5, 50.5]	62.0 [43.5, 71.5]	0.236

**Table 3:** Non-cumulative median CCI [IQR] for all rehospitalizations at <3, <6, <12, and >12 months after transplantation.

Variable	ABO Compatible	ABO Incompatible	*p*-Value
CCI < 3 months	33.7 [28.2, 37.5]	31.4 [20.9, 35.2]	0.438
CCI < 6 months	33.7 [29.9, 35.6]	33.7 [33.7, 39.6]	0.875
CCI < 12 months	33.1 [23.7, 38.2]	20.9 [20.9, 31.7]	0.25
CCI > 12 months	31.9 [24.7, 39.1]	27.1 [23.2, 34.3]	0.163

## Data Availability

The data presented in this study are available upon request from the corresponding author. Due to privacy reasons as well as ethical restrictions, data cannot be available publicly.

## Abbreviations

ABOi	ABO incompatible
ABOc	ABO compatible
ABOi-LDKT	ABO-incompatible live-donor kidney transplantation
ABOc-LDKT	ABO-compatible live-donor kidney transplantation
ABOi-LDKTr	ABO-incompatible live-donor kidney transplant recipient
ABOc-LDKTr	ABO-compatible live-donor kidney transplant recipient
ACS	acute coronary syndrome
CCI	comprehensive complication index
CKD-epi	Chronic Kidney Disease Epidemiology Collaboration
DDKT	deceased donor kidney transplantation
DGF	delayed graft function
DSA	donor-specific antibodies
eGFR	estimated glomerular filtration rate
ESRD	end-stage renal disease
KPD	kidney paired donation
KT	kidney transplantation
LDKT	living donor kidney transplantation
MFI	mean fluorescent index
NODAT	new-onset diabetes after transplantation
